# ECDC expert opinion on efficacy and effectiveness of neuraminidase inhibitors published for public consultation

**DOI:** 10.1111/irv.12377

**Published:** 2016-03-31

**Authors:** Pasi Penttinen, Mike Catchpole

Neuraminidase inhibitors (NAI) are generally recommended for patients with severe or progressive influenza requiring hospitalisation in Europe. According to a recent survey by the VENICE^1^ initiative funded by the European Centre for Disease Prevention and Control (ECDC), national recommendations regarding influenza antiviral use are available in 24 EU/EEA Member States. These recommendations are generally in line with ECDC guidance developed for the 2009 pandemic.^1^ Fourteen EU/EEA Member States also recommend use in residents of nursing homes or other long‐term care facilities at risk of severe disease. Only a minority of EU/EEA Member States recommend antivirals as treatment or prophylaxis for outpatients who have a higher risk of severe outcomes of influenza (young children, elderly or individuals of any age with underlying chronic illnesses).

Oseltamivir and zanamivir, the NAIs currently authorised in the EU for treatment and prophylaxis of seasonal, pandemic and zoonotic influenza disease, have been subject to a heated public debate in recent years concerning their effectiveness and safety profile,^2^, ^3^, ^4^ as well as the appropriateness of stockpiling these drugs for use in future influenza pandemics. This debate was rekindled in the last year by the work and the public statements of the Cochrane collaborations acute respiratory infections group and the publicity given to this by the British Medical Journal, with articles and editorials in the journal and accompanying content on the BMJ website.^5^

The debate has left many clinicians and public health practitioners confused about the role of NAIs in treatment and prophylaxis of influenza, and many public health experts believed that potential public health benefits were being missed as a consequence. In the light of this, the ECDC Advisory Forum, consisting leading independent public health experts from EU Member States, requested an assessment of the evidence of the efficacy and effectiveness of NAI's for public health use in influenza outbreak settings, specifically during institutional outbreaks and new and emerging influenza virus outbreaks. Further, in August 2014, the EU Health Security Committee requested a review of the evidence by ECDC. Hence, an expert consultation with an international group of public health experts was convened by ECDC in Stockholm, in February 2015, to review data presented in newly conducted systematic reviews/meta‐analyses of clinical studies on influenza antivirals and to develop an ECDC Expert Opinion.

Three recent large meta‐analyses assessing effectiveness and safety of the two licensed neuraminidase inhibitors, oral oseltamivir and inhaled zanamivir, were reviewed: The 2014 Cochrane Collaboration report (Jefferson *et al*.),^6^ the 2015 MUGAS study (Dobson *et al*.) 7 and the 2014 PRIDE study (Muthuri *et al*.).^8^ Jefferson *et al*. and Dobson *et al*. reviewed randomized, placebo‐controlled trials that enrolled outpatients <48 hours since illness onset (Dobson *et al*. did an individual patient‐level analysis), and Muthuri *et al*. included data from observational studies that enrolled patients hospitalised with H1N1pdm09. Additional reviews and studies were considered where appropriate.

Many of the trials included in the reviews by Jefferson *et al*. and Dobson *et al*. were conducted among previously healthy adult populations, whereas much of the recommended use of NAIs for outpatients is in respect of decreasing the risk of severe outcomes among risk groups. Treatment is generally recommended for hospitalised influenza patients; however, there is a lack of randomised trials in this important subpopulation. This imbalance between the focus of policy and the focus of trials providing evidence for policy is problematic. However, public health recommendations are needed. It was in the face of this disconnect between the published evidence base and the real‐world policy questions that ECDC chose to review and complement the published evidence with the expert opinion of a multinational and multidisciplinary expert group.

The reviews by Jefferson *et al*. and Dobson *et al*. conclude that, for adults, oseltamivir decreases the time to *first* alleviation of symptoms of influenza‐like illness (ILI) by 16·8 hours (95% CI 8·4–25·1) and the time to alleviation of *all* symptoms by 25·2 hours (95% CI 16·0–36·2), respectively.

It should be noted that many of the original trials on oseltamivir included in the reviews by Jefferson *et al*. and Dobson *et al*. were underpowered to study severe outcomes, as these were not the primary outcomes of interest in those studies. Nonetheless, additional analyses within the Jefferson *et al*. and Dobson *et al*. reviews suggest beneficial effects on lower respiratory tract complications including pneumonia, hospitalisations and severe outcomes (patients receiving intensive care or cases of death). Many of these effects could only be evaluated from the results of RCTs with more than 1000 individuals (estimated hospitalisation rate <1% of all infected) in the intervention arms or in observational studies.

The lack of sufficiently powered trials, especially among the severely ill and hospitalised patients, means that the recommendations on the use of NAIs necessarily have to rely substantially on observational studies. In the pooled individual data from observational studies on hospitalised influenza patients, analysed by Muthuri *et al*., and reviewed by the ECDC expert panel, an effect on mortality was observed. During the three pandemic waves of the influenza A(H1N1)pdm09 in 2009–2011, decreased mortality was associated with the use of neuraminidase inhibitors among hospitalised patients (OR 0·81; 95% CI 0·70–0·93). After controlling for timing of treatment, this association was more pronounced.

Several additional, more recent, randomised control trials and large observational studies were identified during this expert consultation, further supporting the evidence of effectiveness among specific risk groups. For example, oseltamivir treatment has been assessed from registry data in patients ≥18 years with an already known cardiovascular disease.^9^ The incidence of recurrent cardiovascular events within 30 days after the influenza diagnosis was significantly reduced (OR 0·42; 95% CI 0·35‐0·50) in the treatment group. Further, a systematic review conducted in 2011 on five observational studies on pregnant women reported that neuraminidase inhibitors administered within 48 hours from onset of symptoms compatible with influenza conferred decreased risk of severe disease.^10^ Observational studies are prone to bias and confounding, which can be minimised through careful study design and adjustment for confounders; therefore, the strength of evidence provided by these studies can vary based on the quality of the study design.

The review of the evidence by the external expert panel convened by ECDC did not identify any significant *new* evidence against current policy on use of neuraminidase inhibitors in most EU/EEA Member States. Further, these recommendations are endorsed by the expert opinion based on this recent review of evidence by the international panel convened by ECDC. This position is also consistent with guidance from the World Health Organization (WHO) and many national public health organisations in North America, South East Asia, Australia, Japan and New Zealand.

The expert panel also concluded that further studies are needed on current neuraminidase inhibitors authorised within the EU/EEA and elsewhere, as well as development of further influenza antivirals to protect the EU/EEA population. The evidence for currently authorised neuraminidase inhibitors in the EU/EEA needs to be expanded in the knowledge of more rare but severe endpoints such as reduction in mortality, intensive care including mechanical ventilation and ECMO treatment and long‐term sequelae.

While the expiry of the patent of oseltamivir will likely make the medicine more affordable to patients and healthcare systems in the near future, more effective antiviral drugs to decrease morbidity and severe outcomes of influenza infection would be beneficial. Research and development work is underway on several new antivirals, alone or in combination with current drugs.

The draft expert opinion is now out for public consultation on the ECDC Website [link], and ECDC welcomes all input on the document.

## Note

1 The Vaccine European New Integrated Collaboration Effort.
